# Navigating grey areas in HIV and mental health implementation science

**DOI:** 10.1002/jia2.26271

**Published:** 2024-06-25

**Authors:** Audrey Harkness, Ali Giusto, Alison B. Hamilton, Raul U. Hernandez‐Ramirez, Donna Spiegelman, Bryan J. Weiner, Rinad S. Beidas, Michaela E. Larson, Sheri A. Lippman, Milton L. Wainberg, Justin D. Smith

**Affiliations:** ^1^ School of Nursing and Health Studies University of Miami Coral Gables Florida USA; ^2^ Department of Psychiatry Columbia University Irving Medical Center New York State Psychiatric Institute New York New York USA; ^3^ Center for the Study of Healthcare Innovation Implementation & Policy, VA Greater Los Angeles Healthcare System Los Angeles California USA; ^4^ Department of Psychiatry & Biobehavioral Sciences University of California Los Angeles Los Angeles California USA; ^5^ Department of Biostatistics Center for Interdisciplinary Research on AIDS and Center for Methods in Implementation and Prevention Science Yale School of Public Health New Haven Connecticut USA; ^6^ School of Public Health University of Washington Seattle Washington USA; ^7^ Department of Medical Social Sciences Feinberg School of Medicine Northwestern University Chicago Illinois USA; ^8^ Division of Prevention Science University of California San Francisco San Francisco California USA; ^9^ Department of Population Health Sciences Spencer Fox Eccles School of Medicine at the University of Utah Salt Lake City Utah USA

**Keywords:** community, HIV, implementation science, mental health, methods, research design

## Abstract

**Introduction:**

Implementation science (IS) offers methods to systematically achieve the Ending the HIV Epidemic goals in the United States, as well as the global UNAIDS targets. Federal funders such as the National Institutes of Mental Health (NIMH) have invested in implementation research to achieve these goals, including supporting the AIDS Research Centres (ARCs), which focus on high‐impact science in HIV and mental health (MH). To facilitate capacity building for the HIV/MH research workforce in IS, “grey areas,” or areas of IS that are confusing, particularly for new investigators, should be addressed in the context of HIV/MH research.

**Discussion:**

A group of IS experts affiliated with NIMH‐funded ARCs convened to identify common and challenging grey areas. The group generated a preliminary list of 19 grey areas in HIV/MH‐related IS. From the list, the authors developed a survey which was distributed to all ARCs to prioritize grey areas to address in this paper. ARC members across the United States (*N* = 60) identified priority grey areas requiring clarification. This commentary discusses topics with 40% or more endorsement. The top grey areas that ARC members identified were: (1) Differentiating implementation strategies from interventions; (2) Determining when an intervention has sufficient evidence for adaptation; (3) Integrating recipient perspectives into HIV/MH implementation research; (4) Evaluating whether an implementation strategy is evidence‐based; (5) Identifying rigorous approaches for evaluating the impact of implementation strategies in the absence of a control group or randomization; and (6) Addressing innovation in HIV/MH IS grants. The commentary addresses each grey area by drawing from the existing literature (when available), providing expert guidance on addressing each in the context of HIV/MH research, and providing domestic and global HIV and HIV/MH case examples that address these grey areas.

**Conclusions:**

HIV/MH IS is key to achieving domestic and international goals for ending HIV transmission and mitigating its impact. Guidance offered in this paper can help to overcome challenges to rigorous and high‐impact HIV/MH implementation research.

## 1 INTRODUCTION

Implementation science (IS), defined by the National Institutes of Health (NIH) as “the scientific study of the use of strategies to adopt and integrate evidence‐based health interventions (EBIs),” offers methods to achieve the United States Ending the HIV Epidemic (EHE) goals and global UNAIDS targets [1, 2, 3, 4]. Federal investments in IS research and training [5, 6] include supporting the National Institute of Mental Health (NIMH) AIDS Research Centres (ARCs), which focus on addressing HIV in the context of mental health (MH) and training the next generation of HIV scientists.

As a relatively new and evolving field, efforts are underway to harmonize IS definitions and approaches [5, 7–10]; however, there are “grey areas” or common points of confusion that present barriers to high‐impact MH/HIV IS [5, 11]. Grey areas present a particular challenge for early stage investigators and those new to the field [12]. This commentary highlights six grey areas in HIV/MH IS and guidelines for navigating them, drawing on the HIV/MH literature and illustrative examples beyond HIV/MH when needed.

Nine ARC‐affiliated IS experts convened via an online meeting and identified 19 grey areas in HIV/MH IS. This informed a survey distributed via listserv through seven ARCs. ARC members across the United States (*N* = 60) completed the survey to prioritize grey areas (i.e. ≥40% endorsement; Table 1). The commentary discusses priority topics and provides guidance on how to navigate them based on existing literature and author expertise. Table 2 provides examples alongside each grey area.

**Table 1:** Results of implementation science grey areas survey of AIDS Research Centre (ARC) members

Grey area^a^	Total *N* = 60
	*n* (%) Endorsed
Determining whether something is an implementation strategy or an intervention	38 (63.3%)
Addressing innovation in implementation science grants	35 (58.3%)
Rigorously evaluating implementation strategies in the absence of a control group^b^	28 (46.7%)
Determining how much evidence there needs to be before an intervention is considered for adaptation	28 (46.7%)
Rigorously evaluating implementation strategies in the absence of randomization^b^	26 (43.3%)
Fitting recipient perspectives in implementation science frameworks, theories and models	26 (43.3%)
Establishing that an implementation strategy is evidence‐based	24 (40.0%)
Identifying the right conditions for doing a hybrid implementation‐effectiveness trial	19 (31.7%)
Establishing that a supporting intervention (i.e. an intervention that is meant to support the implementation of another intervention) is evidence‐based	19 (31.7%)
Defining the role of implementation science in developing practice‐based evidence	19 (31.7%)
Deciding between an implementation trial or hybrid effectiveness‐implementation trial (Type I, II or III) for a given project	19 (31.7%)
Defining “adoption” for interventions where it is the recipient, not the healthcare system, doing the adopting (e.g. direct to consumer digital health interventions)	18 (30.0%)
Deciding when to include implementation components in efficacy trials	18 (30.0%)
Evaluating and distinguishing fidelity to a clinical intervention versus fidelity to an implementation strategy	16 (26.7%)
Distinguishing implementation facilitators (identified through determinants frameworks) from implementation strategies	15 (25.0%)
Identifying the role of observational data in implementation science	17 (28.3%)
Specifying relationships between implementation strategies (e.g. some strategies need other implementation strategies to facilitate their implementation; some implementation strategies must be paired with others to be useful)	15 (25.0%)
Differentiating implementation science and practice	14 (23.3%)
Identifying the right respondents for assessing implementation outcomes (e.g. consumers vs. implementers)	13 (21.3%)

*Note*: *N* = 60 ARC members, including 87% (*n* = 52) of which were early stage investigators or mentors to early stage investigators.

^a^ The survey informed ARC members that the authors were preparing a manuscript to provide practical guidance and strategies for navigating grey areas in implementation science and HIV/MH research. They were then presented with the list of 19 grey areas shown above and asked to select up to eight topics that they felt would be most important to address in this manuscript. “Endorsement” reflects that a respondent indicated that this grey area would be important to address within the manuscript.

^b^ These two grey areas were combined for this manuscript due to conceptual overlap.

**Table 2:** Grey area case examples and recommendations

Grey area	Case example
1. Distinguishing between an implementation strategy versus an intervention	The 2022 special issue of JAIDS [13] highlighted findings from CFAR/ARC Ending the HIV Epidemic Supplement projects and required all submissions to complete a table in which the authors of an implementation study were required to specify the evidence‐based intervention and the implementation strategies and to complete an Implementation Research Logic Model [14] that distinguished the intervention(s) and implementation strategies. This specification helps researchers and practitioners have clarity about what the strategy versus intervention is. For example, within this special issue, Garner and colleagues [15] identified psychosocial (e.g. cognitive‐behavioural therapy) and pharmacological evidence‐based interventions (e.g. naltrexone) for addressing substance use disorders in HIV service organizations. Their goal was to identify interventions that met the needs of these potential implementing organizations. They clearly differentiated these interventions from implementation strategies to support the intervention's implementation and sustainment in their Implementation Research Logic Model (e.g. provide ongoing implementation consultation, provide access to training modules). HIV/MH investigators can navigate grey area #1 by using this type of template, and/or the Implementation Research Logic Model, to provide greater clarity regarding their strategy(ies) versus intervention(s).
2. Determining how much evidence is needed before an intervention can be adapted	In a hybrid type II implementation‐effectiveness study [16], Hamilton and colleagues originally proposed an adapted version of the evidence‐based “Eban” intervention that had been tested and found efficacious for reducing behaviours that can lead to HIV/STD (e.g. condomless sex) in a cluster RCT. Eban is a behavioural intervention delivered to couples that focuses on building self‐efficacy, social support, sexual communication skills, grounded in social cognitive theory and an Afrocentric paradigm [17]. Anticipating challenges with implementation in community‐based HIV service organizations, the investigators proposed to reduce the number of sessions from the original 8‐session design, but this was seen by NIH reviewers as too much of a departure from the evidence base, so the adaptation was not made prior to funding. In conducting the hybrid study, several adaptations did have to be made, particularly in light of the profound influence of external context [18] on implementation. In revisiting Browson and colleagues’ questions about the sufficiency of evidence [19], several conditions were present that supported real‐time adaptation: a pressing health issue (preventing HIV seroconversion in serodiscordant couples), established health equity issues, pressing social and structural determinants, action being taken regardless of the intervention and significant consequences of not implementing, and considering that the participating agencies and clients wanted the intervention (on their terms). The adapted intervention achieved some favourable effectiveness outcomes [20] and patient satisfaction with skills and knowledge gains [21].
3. Fitting recipient perspectives into IS frameworks, theories and models	US and Kenyan researchers collaborated on a recent study to understand barriers and facilitators to delivering a mental health and alcohol use intervention for fathers in Kenya [22]. To identify determinants, a qualitative approach was used that included recipient perspectives. The Integrated Sustainability Framework (ISF) [23] and the Consolidated Framework for Implementation Research (CFIR) [24] guided interviews and analysis of these perspectives. In this case, recipient perspectives were incorporated by interviewing specific individuals who could be impacted by delivery of such a treatment about their attitudes, preferences and beliefs about treatment implementation. Interviews were done with individuals at the patient (i.e. recipient), provider and setting levels, as well as recipients who had previously engaged in the intervention. For instance, fathers experiencing issues addressed by the intervention were interviewed as well as fathers who had previously participated in the specific treatment. Providers, peer‐father counsellors, community leaders, hospital leaders and policy makers were all also interviewed to understand implementation determinants. Recipient perspectives analysed with the framework method identified barriers and facilitators across domains. Barriers included mental health and alcohol use stigma, masculine norms, service cost and alcohol dependence. Facilitators included community buy‐in, family support, providers with lived experience and relevant treatment content (e.g. include content considering masculine norms). Findings are informing implementation strategy development that will continue to include recipient perspectives to finalize strategies. From there, recipient perspectives will be incorporated in the piloting of the intervention and its delivery guided by community‐engaged methods [25] and the RE‐AIM planning and evaluation framework [26].
4. Establishing that an implementation strategy is evidence‐based	Eshun‐Wilson and colleagues [27] conducted a network meta‐analysis to assess the effectiveness of different implementation strategies for distributing HIV self‐testing kits. In this study, the self‐testing kit distribution strategies are the implementation strategies and the HIV self‐testing kits themselves are the intervention. The outcome of interest in the studies included in this meta‐analysis was uptake of HIV testing (i.e. reach, an implementation outcome). The study found that in sub‐Saharan Africa, the most effective self‐testing distribution strategy was through sexual partners, whereas in North America, Asian and Pacific regions, the most effective strategy was web‐based and mail delivery of self‐testing kits. In other words, these distribution strategies (i.e. implementation strategies) were evidence‐based for improving the reach (i.e. implementation outcome) of HIV testing (i.e. intervention) in each context. As another example more specific to MH/HIV, Garner and colleagues [15] created two separate clinicaltrials.gov registrations: one for their effectiveness outcomes linked to the psychosocial intervention to address substance use in HIV organizations [28] and one for their implementation outcomes associated with the two implementation strategies they are comparing in their trial [29]. Their clear reporting of implementation outcomes (e.g. implementation effectiveness index score, level of sustainment score) articulates how the study team will evaluate whether the implementation strategies under study are evidence‐based.
5. Evaluating an implementation strategy's impact in the absence of a control group or randomization	Ruiz and colleagues [30] used a controlled interrupted time series (ITS) study based upon surveillance data from Philadelphia (1984–2015) and Baltimore (1985–2013) to assess the impact of policy changes (i.e. an implementation strategy) allowing implementation of syringe exchange programmes (SEP; in 1992 in Philadelphia; in 1994 in Baltimore) on injection drug use (IDU)‐associated HIV diagnoses. HIV diagnosis data among men who have sex with men (MSM) without IDU exposure was used as a control in Baltimore as the SEP's policy change was not expected to have an impact on that group. The study observed a decrease in the number of IDU‐associated HIV diagnoses after legal SEP implementation. In Philadelphia, legal SEP implementation in 1992 resulted in both an immediate significant 163 fewer new IDU‐associated HIV diagnoses along with a continued fewer new 116 cases/year between 1993 and 2015 (*p*<0.001 for both). In Baltimore, only a borderline significant fewer new 83 cases per year occurred between 1995 and 2013 (*p* = 0.09), with no further significant immediate decrease after legal SEP implementation in 1994. Among MSM in Baltimore, no statistically significant decrease or change over time in HIV diagnoses was observed after legal SEP implementation, suggesting that the change in IDU‐associated HIV diagnoses observed in Baltimore in response to the policy could be causally attributed to the legal SEP utilization. The results may have been influenced by variations of SEP implementation, with Baltimore having a more restrictive policy during part of the post‐implementation period. Using an ITS design, this study provided evidence supporting the effectiveness of policy change as a strategy to enable implementation of evidence‐based SEP to prevent HIV among people who inject drugs.
6. Addressing innovation in IS grants	As part of the Ending the HIV Epidemic plan, the NIH has issued a number of calls for administrative supplements which identify areas of innovation in HIV/MH research that are considered high priority [31]. Projects funded under this initiative innovated by integrating equity into existing IS tools that did not previously have an explicit focus on equity (e.g. equity‐focused implementation mapping; PI: Wilson), implementing status‐neutral HIV linkage services into behavioural health (i.e. mental health/substance use) contexts (i.e. a new approach—status neutral and a new context—behavioural health; PI: Chwastiak) and integrating behavioural health and HIV treatment re‐engagement using data science approaches not typically used in implementation research (PI: Dambrowski). Although all the funded EHE supplements have innovated in HIV/MH IS, these are some examples of how to describe innovation in these types of grants. In addition, we emphasize that adapting and/or scaling up or out evidence‐based implementation strategies and/or interventions is itself an innovative IS activity.

Abbreviations: ARC, AIDS Research Centre; CFAR, Centre for AIDS Research; EHE, Ending the HIV Epidemic; IDU, injection drug use; IS, implementation science; JAIDS, Journal of Acquired Immune Deficiency Syndromes; MH, mental health; MSM, men who have sex with men; NIH, National Institutes of Health; RCT, randomized controlled trial; RE‐AIM, Reach, Effectiveness, Adoption, Implementation, Maintenance/Sustainment; SEP, syringe exchange programme; STD, sexually transmitted disease.

## 2 DISCUSSION

### 2.1 Grey Area 1: What is an implementation strategy and what is an intervention?

Despite the centrality of implementation strategies to IS, there are few practical resources to distinguish implementation strategies from HIV/MH interventions [7]. Interventions are “programmes, practices, principles, procedures, products, pills, or policies that have been demonstrated to improve health behaviours, health outcomes, or health‐related environments” [32]. The definition of implementation strategies—“actions taken to enhance adoption, implementation, and sustainability of EBIs” [32]—underscores the need to first define the health‐related intervention. For example, pre‐exposure prophylaxis (PrEP) is a health intervention that prevents HIV. Implementation strategies to improve the reach of PrEP to populations affected by MH concerns might include provider training, integrating PrEP delivery in MH settings or community‐based PrEP distribution [33]. Patients/clients are the direct recipient of interventions, whereas implementation strategies are commonly (but not always) directed towards implementers and delivery systems/organizations. Recently, a third category—adjunctive interventions—has been proposed, which are behaviour change interventions that target recipients (patients/clients) to support their use of a health intervention [34]. For instance, peer navigation to increase uptake of PrEP and ancillary MH services would be considered an adjunctive intervention under this definition.

A common source of confusion in the IS field is whether implementation strategies can be directed to clients/patients. The Expert Recommendation for Implementing Change (ERIC) compilation defined several recipient (client/patient)‐directed engagement strategies. For example, medication adherence counselling is a recipient engagement strategy to increase the use of an evidence‐based intervention (e.g. antiretroviral therapy, anti‐depressants). Based on Smith and colleagues’ guidelines [34], adherence counselling would be considered an adjunctive intervention. However, there are also recipient‐directed strategies that would be considered implementation strategies, per the same guidelines [34]. For instance, the PrEP4Love campaign [35] could be considered an implementation strategy that is population‐focused (i.e. not just directed towards those already engaged in the intervention) to increase awareness and interest in PrEP.

Smith et al. [34] provide a practical tool (a decision tree) for distinguishing interventions from implementation strategies in HIV/MH research based on these questions: (1) Will this intervention/strategy be directly responsible for an HIV/MH health outcome (e.g. viral suppression, prevention of HIV acquisition, reduction of MH symptoms). If yes, this is a health intervention; and (2) Does this intervention/strategy lead to another outcome that precedes the HIV/MH health outcome (e.g. offering PrEP navigation to recipients or training HIV test counsellors in PrEP navigation)? If yes, this could be either an adjunctive intervention or an implementation strategy depending on function. Responses to these questions may depend on the context and function of the intervention/strategy. For instance, training peers to deliver an MH/HIV‐prevention programme could be considered a task‐shifting implementation strategy. However, to the extent that training the peers is also intended to improve their own MH/HIV health outcomes, it could also be considered an intervention.

Strategies are often tested as interventions. Several “interventions” in the CDC Compendium of Evidence‐Based Interventions/Best Practices for HIV Prevention [36] are or can also be implementation strategies (e.g. “interventions” that target system change to improve linkage to HIV treatment). Testing strategies explicitly as strategies facilitates studying the process, procedures and context of the activity or approach, which may be overlooked when evaluating a strategy as an intervention as noted by Smith et al. [34]. Additionally, multilevel interventions can contain interventions directed to recipients and strategies targeting other levels (e.g. clinicians, leadership). For example, King et al. evaluated social/sexual network distribution (implementation strategy) of HIV self‐testing kits (intervention) [37]. In this example, it is essential to distinguish each element as an intervention or strategy, as the authors did, while still testing the multilevel intervention collectively. Testing a strategy that is misclassified as an intervention means overlooking proximal implementation outcomes (e.g. reach of self‐testing kits) in favour of distal health outcomes (e.g. knowledge of HIV status), therefore, limiting IS knowledge contributions and inaccurately assuming the strategy alone was responsible for health outcomes. Using the Implementation Research Logic Model [14] can be helpful in determining the function of interventions and strategies and appropriately designing studies and evaluation plans [34, 38]. For more discussion of this grey area, see Smith et al. [34].

### 2.2 Grey Area 2: How much evidence is needed to consider an intervention for adaptation?

An IS grey area that is highly relevant to HIV/MH IS is how much evidence is needed before adapting an intervention. The Adaptome [39] posits five sources of adaptations: service setting, target audience, delivery model, cultural and core components. IS views adaptation as inherent to implementation and suggests adapted interventions “borrow strength” from prior trials [40]. A goal of the EHE plan, and IS in general, is to shorten the time between demonstrating an intervention's efficacy and implementation. Requiring randomized controlled trials (RCTs), which are the traditional litmus test for whether an intervention is appropriate for adaptation, could impede this goal [41]. There are circumstances in which one may wish to adapt an EBI for implementation without gold standard evidence; in fact, some have suggested considering more flexible standards that acknowledge that evidence is not static, nor neutral [39]. New analytic techniques are being explored to evaluate adaptations to interventions during implementation, which could strengthen the evolving evidence base [42]. Implementation scientists have recommended rigorous alternatives to RCTs (grey area 5) and sometimes skipping efficacy trials in favour of effectiveness trials and hybrid effectiveness‐implementation studies [43, 44]. For example, Hamilton and colleagues [45] are conducting a hybrid effectiveness‐implementation study testing a blended intervention (not tested in a prior RCT) that draws upon several EBIs to address the needs of people living with HIV who have histories of trauma and cardiovascular risk.

Several considerations can help investigators navigate this grey area. These include considering stakeholder priorities, equity and urgency or consequences of not implementing when deciding whether evidence is sufficient to adapt an intervention [19], and ensuring some benefits—and no harms—have been demonstrated prior to adaptation [46]. Investigators can also assess the intervention's (1) accessibility to potential implementers, (2) mechanism of change and fit for the population, and (3) acceptability for the population [47]. These considerations do not suggest that evidence for an intervention is unnecessary, but that investigators should weigh them with the evidence for the intervention [46]. As a case example of this decision‐making process, Marc and colleagues [48] applied a rapid implementation process to identify “evidence‐informed” interventions to improve HIV outcomes among populations disproportionately affected by HIV. They developed scoring criteria that weighed factors including research evidence, feasibility, cultural appropriateness and context to identify evidence‐informed interventions that they then adapted for local implementation. HIV/MH IS researchers may find it useful to draw on their scoring criteria to decide whether to adapt an existing intervention (e.g. to complete the “decide” step of ADAPT‐ITT, a widely used adaptation framework [49]).

### 2.3 Grey Area 3: Where do recipients’ perspectives “fit” into IS frameworks, theories and models?

Recipient perspectives—the beliefs, preferences and experiences of individuals or groups who receive interventions—are key to enhancing the relevance, acceptability and effectiveness of HIV/MH interventions and the strategies selected for delivery. Recipients can inform the selection of what will be implemented, guide EBI adaptation and facilitate understanding of implementation determinants. Some frameworks, theories and models explicitly include recipient perspectives (e.g. the updated Consolidated Framework for Implementation Research [50]), but some do not. IS is bringing renewed focus to integrating recipient perspectives using approaches like community‐based participatory research, implementation mapping [51] or user‐centred design [52, 53].

HIV/MH interventions may be underused, or used inequitably, if they are not accessible, acceptable or responsive to the needs of people affected by HIV in ways that consider intersectionality. As a result, reach is an implementation outcome of focus, typically measured at the level of the recipient, utilized to understand whether implementation strategies for HIV/MH interventions improve access to and use of those interventions for underserved, minoritized or discriminated against populations experiencing HIV or MH healthcare disparities [54, 55]. Recipient perspectives are central to improving reach, accessibility, acceptability and ensuring equitable implementation of EBIs to redress health inequities.

While retaining focus on recipients in HIV/MH IS, it is also important to note that recipients are not the only focus of HIV/MH IS. Though many HIV researchers have traditionally focused exclusively on recipients, it is important to address multilevel (e.g. provider, organizational, structural) implementation determinants [33, 56]. Reviews of HIV IS research suggest a need to integrate implementer/implementing system perspectives to a greater extent, alongside recipient perspectives.

### 2.4 Grey Area 4: What does it take to establish that an implementation strategy is evidence‐based?

HIV/MH researchers have extensive experience demonstrating the efficacy and effectiveness of interventions, but less with rigorously evaluating and testing implementation strategies [57]. Evidence about the degree to which strategies impact implementation outcomes is needed to inform implementation practice and achieve EHE goals [58, 59].

When designing and reporting on a study meant to evaluate the evidence for an implementation strategy, it is important to first define the intervention(s) and implementation strategy(ies) (grey area 1). Linked to this determination, the distinction between implementation and efficacy/effectiveness trials is that in an implementation trial, implementation outcomes (e.g. peer deliverers’ fidelity to an MH/HIV prevention programme) associated with an implementation strategy (e.g. weekly supervision from a licensed psychologist) must be assessed [10]. An effectiveness trial evaluates health outcomes (e.g. prevention of HIV acquisition, reduced depression symptoms) associated with the intervention. The relationship between the two is that implementation strategies that positively impact implementation outcomes should improve the effectiveness of interventions.

To reduce confusion, implementation studies evaluate outcomes at a different level than efficacy/effectiveness studies which evaluate interventions. For instance, in a hybrid study evaluating the effectiveness of an intervention (e.g. a substance use treatment programme to prevent HIV acquisition) and an implementation strategy (e.g. train‐the‐trainer to enhance adoption of the treatment programme in clinical settings), investigators should specify what evidence the trial provided regarding effects of the (1) intervention (i.e. clinical outcomes) and (2) implementation strategy (i.e. implementation outcomes such as adoption of an EBI, fidelity to the EBI, organizational sustainment of an EBI).

### 2.5 Grey Area 5: How can a rigorous evaluation of implementation strategies be done without a control group or randomization?

To conduct causal inference testing about the impact of implementation strategies, it is necessary, but not sufficient, that a comparison group be available [60]. When conducted properly, RCTs are the gold standard for causal inference [61]; randomization ensures, on average, the comparability/exchangeability between groups [62], to ensure that the potential outcomes are independent of the intervention received [63]. However, causal inference can be determined using other rigorous methods that may be more acceptable to community partners. If implementation strategy evaluations were restricted exclusively to randomized trial designs, knowledge about their impact would be severely restricted [64]. Thus, researchers must consider non‐randomized designs [46, 65–68], accompanied by rigorous analytic methods to strengthen internal and external validity. Non‐randomized designs are underused in NIH‐funded HIV IS [69], limiting the knowledge base about the utility and contextualization of implementation strategies.

A simple quasi‐experimental approach is the pre‐post design [65, 66, 70–72]. This design yields causal effect estimates when no background time effects exist. A pre‐post design, with a comparison group, can provide more causally rigorous estimates than a standard (uncontrolled) pre‐post design, because it requires before‐after data in comparable control settings/individuals [65, 66, 73–76]. When outcomes are repeatedly measured over time before and after implementation, the interrupted time series (ITS) design can be used [66, 77] to assess changes and trends in outcomes after introducing strategies [78 ^,^ 79] at a clearly defined time point at one or more sites. ITS designs can include control settings [68, 80–82] and randomization [83], strengthening causal inference by controlling for background time effects. When the strategy needs to be delivered to all participants or cannot be rolled out at the same time to everyone (individuals or clusters), using a stepped wedge design (SWD) or other rollout design to introduce the intervention over several staggered time periods after an initial baseline period may be desirable [84]. Here, the intervention start time for each group is usually randomized. SWDs can include cohort and repeated cross‐sectional designs [66], and permit both before‐after and between‐groups comparisons, reducing confounding bias by time‐invariant and time‐varying factors [84]. Given the need for data at multiple times and/or for a long duration, ITS and SWD may be time‐intensive and costly, but can be facilitated by automated or existing sources for data collection [66].

Causal inference methods also permit the generation of evidence in non‐randomized observational research [60, 85]. To successfully apply these methods, data on the implementation strategies used and implementation and/or clinical outcomes, and all potential confounders, effect modifiers and mediators of their associations must be available. Using these methods, case‐control and cohort study designs can be used to causally evaluate impact [65, 86]. Additional approaches include restriction, matching [66, 87], synthetic controls [88], standard multivariable modelling, marginal structural models, propensity score weighting and nested structural failure time models [60] to control confounding [87] by individual and contextual factors to obtain causal estimates.

### 2.6 Grey Area 6: What does “innovative” mean in IS grants?

Successfully competing for NIH and other funding requires researchers to explain how their research innovates from current research, clinical approaches, methods, theories, or otherwise shifts beyond current practice. This can be challenging in IS because the proposed EBIs are often not new, nor are the strategies, frameworks, designs or methods.

To address this, investigators can regard innovation not as something new to the world but as a departure from the status quo that could overcome the limitations of current approaches and advance HIV/MH research or practice [89]. Articulating innovation in an IS grant requires making the case that the study will open new horizons, options or directions, or advance the science of implementation. If the status quo is characterized by effectiveness research, asking implementation questions can be innovative. Within IS, under‐researched topics (e.g. evaluating strategies for sustaining HIV/MH interventions) and “frontier” topics (e.g. optimizing implementation strategies for effectiveness and affordability, mechanisms) shift the science in new directions. If the research questions or topics are not novel, consider aspects of the approach that could address methodological limitations of prior research that have stifled progress in HIV/MH IS. For example, the deployment of a commonly used implementation strategy (e.g. integrated MH care) in a novel context (e.g. HIV clinics) could overcome the limited success of previous approaches and expand service delivery options. Application of IS frameworks that focus on equity [90, 91] or include contextual features distinctive in low‐ or middle‐income countries [92] could advance HIV/MH implementation research by redirecting scientific inquiry to novel implementation strategies, novel doses of these strategies or combinations of strategies. Proctor and colleagues [93] and Crable and colleagues [94] provide additional guidance for addressing this grey area.

## 3 CONCLUSIONS

Achieving domestic and global HIV targets requires rigorous IS research and, therefore, necessitates navigating and clarifying grey areas affecting HIV/MH IS. This commentary underscores the importance of distinguishing implementation strategies from interventions, embracing complexity when determining whether interventions are “ready” for adaptation, balancing recipients’ and implementers’ perspectives into IS, operationalizing and testing the effectiveness of implementation strategies, leveraging non‐randomized designs to enhance HIV/MH IS and reconceptualizing what it means to be “innovative” in HIV/MH IS grant applications.

## COMPETING INTERESTS

RSB is the principal at Implementation Science & Practice, LLC. She is currently an appointed member of the National Advisory Mental Health Council and the NASEM study, “Blueprint for a national prevention infrastructure for behavioural health disorders,” and serves on the scientific advisory board for AIM Youth Mental Health Foundation and the Klingenstein Third Generation Foundation. She has received consulting fees from United Behavioral Health and OptumLabs. She previously served on the scientific and advisory board for Optum Behavioral Health and has received royalties from Oxford University Press. All activities are outside of the submitted work.

## AUTHORS’ CONTRIBUTIONS

Contributions to this manuscript were as follows: Conceptualization (AH, ABH, RUHR, DS, BJW, RSB, SAL, MLW and JDS), Project Administration (AH and MEL), Writing—original draft (AH, AG, ABH, RUHR, DS, BJW, and JDS), Writing—review and editing (AH, AG, ABH, RUHR, DS, BJW, RSB, MEL, SAL, MLW and JDS).

## FUNDING

This project was supported by several grants, including K23MD015690 (PI: Harkness), P30MH116867, P30MH133399 (PI: Safren), P30MH123248 (PI: Simoni), P30AI117943 (PI: D'Aquila), T32MH096724 (PI: Wainberg), D43 TW011302 (MPI: Sohn, Wainberg), P30MH062246 (PI: Johnson), R01NR019753 (MPI: Momplaisir, Beidas, Gross), P30MH058107‐25S2 (PI: Hamilton), U01HL142109 (MPI: Wyatt, Brown, Hamilton), VA HSR&D RCS 21–135 (PI: Hamilton), VA QUERI QUE 20–028 (PI: Hamilton, Bean‐Mayberry, Farmer, Moin), VA HSR&D SDR 10‐012 (PI: Yano), P30MH062294 (PI: Kershaw), 1K23MH128742‐01A1 (PI: Giusto) and 2P30MH097488 (Metzger).

## DISCLAIMER

The contents of this manuscript are solely the responsibility of the authors and do not necessarily represent the official views of the NIH.

## Data Availability

Data sharing is not applicable to this article as no datasets were generated or analysed during the current study.
